# Small Molecule Tyrosine Kinase Inhibitors of ErbB2/HER2/Neu in the Treatment of Aggressive Breast Cancer

**DOI:** 10.3390/molecules190915196

**Published:** 2014-09-23

**Authors:** Richard L. Schroeder, Cheryl L. Stevens, Jayalakshmi Sridhar

**Affiliations:** 1 Department of Chemistry, Xavier University of Louisiana, 1 Drexel Drive, New Orleans, LA 70125, USA; E-Mail: rschroed@xula.edu; 2 Ogden College of Science and Engineering, Western Kentucky University, 1906 College Heights Boulevard #11075, Bowling Green, KY 42101, USA; E-Mail: cheryl.stevens@wku.edu

**Keywords:** human epidermal growth factor 2 (HER2), kinase domain, HER2 inhibition, monoclonal antibodies, small molecule inhibitors

## Abstract

The human epidermal growth factor receptor 2 (HER2) is a member of the erbB class of tyrosine kinase receptors. These proteins are normally expressed at the surface of healthy cells and play critical roles in the signal transduction cascade in a myriad of biochemical pathways responsible for cell growth and differentiation. However, it is widely known that amplification and subsequent overexpression of the HER2 encoding oncogene results in unregulated cell proliferation in an aggressive form of breast cancer known as HER2-positive breast cancer. Existing therapies such as trastuzumab (Herceptin**^®^**) and lapatinib (Tyverb/Tykerb**^®^**), a monoclonal antibody inhibitor and a dual EGFR/HER2 kinase inhibitor, respectively, are currently used in the treatment of HER2-positive cancers, although issues with high recurrence and acquired resistance still remain. Small molecule tyrosine kinase inhibitors provide attractive therapeutic targets, as they are able to block cell signaling associated with many of the proposed mechanisms for HER2 resistance. In this regard we aim to present a review on the available HER2 tyrosine kinase inhibitors, as well as those currently in development. The use of tyrosine kinase inhibitors as sequential or combinatorial therapeutic strategies with other HER family inhibitors is also discussed.

## 1. Introduction

Breast cancer is among the most commonly diagnosed cancers worldwide and is the number one cancer found in women, with an estimated 14.1 million cases reported in 2012 [1]. Overexpression of the human epidermal growth factor receptor 2 (HER2) is responsible for nearly 15%–30% of breast cancers, and is generally associated with poor patient survival [1,2,3]. HER2 is a 185-kDa transmembrane protein encoded by the erbB2 oncogene located on chromosome 17q21-22 [1]. It is a member of the erbB class of receptor tyrosine kinases consisting of four homologous proteins: HER1 (EGFR/ErbB1), HER2 (ErbB2/Neu), HER3 (ErbB3), and HER4 (ErbB4). Normal expression of these proteins at the cell surface is essential for regulating cell growth and epithelial cell survival. However, amplification and overexpression of the HER2 oncogene is typically seen in aggressive metastatic breast cancer, though it is also observed in other malignancies as well [4]. In addition to neglected locally advanced cancers and metastases of low-stage breast cancer, factors such as the rate of proliferation or the ER status of the tumor are related to the recurrence. It has been suggested that prolonged treatment with tamoxifen and/or longer duration of treatment are better at reducing recurrence and prolonging survival than shorter duration [5,6].

Structurally, the HER family consists of an extracellular growth factor ligand-binding domain, a lipophilic alpha helical transmembrane region, and a catalytically active intracellular tyrosine kinase domain. Members of the HER family, with the exception of HER2, have been shown to exhibit ligand specificity (Table 1), where ligand binding induces receptor homodimerization or heterodimerization. Receptor dimers allow intracellular autophosphorylation of specific tyrosine residues within the catalytic kinase domain [7]. The activated dimer complex triggers important downstream cell signaling pathways such as mitogen-activated protein kinase (MAPK), phosphatidylinositol-3 kinase (PI3K)/Akt, phospholipase Cγ, and protein kinase C among others [8,9]. There are no known natural ligands that bind to HER2 specifically. HER2 is known to be a preferred dimerization partner forming potent heterodimers with EGFR and HER3 despite its inability for direct ligand interaction [10].

**Table 1:** Known growth factor ligands and their associated erbB receptors.

Growth Factor	EGFR	HER2	HER3	HER4
Amphiregulin	+	−	−	−
Betacellulin	+	−	−	+
Epiregulin	+	−	−	+
Epigen	+	−	−	−
Epidermal Growth Factor (EGF)	+	−	−	−
Heparin-binding EGF-like Growth Factor (HB-EGF)	+	−	−	+
Transforming Growth Factor alpha (TGF-α)	+	−	−	−
Neuregulin-1 (NRG1)	−	−	+	+
Neuregulin-2 (NRG2)	−	−	+	+
Neuregulin-3 (NRG3)	−	−	−	+
Neuregulin-4 (NRG4)	−	−	‒	+

A 100–115 kDa truncated form of HER2 (p95^HER2^) is expressed in nearly 30% of HER2-positive breast cancers and contains a series of carboxy-terminal fragments [11,12]. Despite lacking an extracellular domain, p95^HER2^ still triggers proliferative downstream signaling events and forms potent heterodimers due to its active kinase region [12]. The lack of an extracellular domain prohibits ligand targeting antibody therapies from interacting with the protein and promotes a cause of resistance for such drugs. However, since p95^HER2^ contains an active kinase domain it remains susceptible to kinase inhibition and serves as an important target in overcoming antibody resistant therapies. Small molecule tyrosine kinase inhibitors are covered extensively in the literature [13,14,15,16,17,18,19,20,21], however an emphasis on the recent applications and the developing therapeutic benefits regarding erbB2 inhibition in the last 5–10 years of clinical studies are reported herein.

## 2. Monoclonal Antibodies in HER2 Inhibition

### 2.1. Trastuzumab

Trastuzumab (Herceptin**^®^**) is a humanized IgG1-class monoclonal EGFR antibody inhibitor that prevents erbB hetero and homodimerization by binding to the extracellular domain of EGFR [22]. Trastuzumab therapy has shown to increase patient survival rate in HER2-overexpressed breast cancers, although the precise mechanism of action remains unclear [4,23,24,25,26,27]. As a monotherapeutic agent trastuzumab shows a 15%–26% response rate in HER2-positive breast cancer, with a median duration of 9 months [22]. In clinical benefit rates measuring a stable disease of 6 months or greater in combination with the overall response rate, trastuzumab shows a 36%–48% benefit in HER2-positive patients [22]. When combined with chemotherapy, trastuzumab shows even greater increased overall patient survival, progression-free survival, and higher response rates than as a single treatment alone [27,28,29,30,31,32]. It is of note, however, that many HER2-overexpressing patients do not respond to trastuzumab treatment alone, and almost all patients develop resistance over time. It has been suggested that a better understanding of molecular mechanisms underlying primary or acquired resistance to trastuzumab is essential for exploring alternative therapeutic avenues. The administration of tyrosine kinase inhibitors either alone or in combination with trastuzumab or antibody-drug conjugates have been suggested as viable alternatives [33].

### 2.2. Pertuzumab

Pertuzumab (Perjeta^®^) is a recombinant humanized monoclonal antibody that exhibits a different extracellular binding specificity than trastuzumab, providing a more comprehensive signal blockade than trastuzumab treatment alone. It was granted accelerated approval by the U.S. Food and Drug Administration based on its phase II pathologic complete response rate (pCR) in neoadjuvant treatments of HER2-positive early-stage, inflammatory, and locally advanced breast cancer with trastuzumab and docetaxel [34]. Both arms of a phase IIa trial investigating the first-line treatment of pertuzumab in combination with trastuzumab, capacitabine and cisplatin in HER2-positive advanced gastric cancer found the drugs to be well tolerated, with a partial response achieved by 86% (arm A) and 55% (arm B) of patients [35]. In a double-blind, randomized phase III trial comparing the safety and efficacy of pertuzumab with trastuzumab and docetaxel, the median progression-free survival was 18.5 months; while the overall survival and objective response rate (80.2%) was higher than the observed placebo group, leading the European Medicines Agency to also approve pertuzumab for treating HER2-positive breast cancer [36].

## 3. Small Molecules as HER2 Kinase Inhibitors

The human genome encodes hundreds of protein kinases that share a conserved catalytic domain in sequence and structure, but are very different in catalysis regulation [37]. Small molecule tyrosine kinase inhibitors compete with ATP at the cytoplasmic catalytic kinase domain to prevent tyrosine phosphorylation and signaling events downstream of ligand binding. Due to the conserved kinase structure, many small molecule targeted therapies exhibit a degree of promiscuity and bind to multiple kinases. Thus, maintaining a high selectivity profile is very important in developing new inhibitors. Protein crystal structures and computational molecular modeling and docking studies have been invaluable in the kinase drug design process. Simulations used to identify key structures and restrictions in the binding pockets allow novel compounds to be screened efficiently. These compounds can exhibit high affinity relationships with their therapeutic targets and thus increase the overall productivity of the hit to lead and optimization strategies used in drug development. The kinase domain of the four members of the ERB family of receptors shows a high degree of homology (59%–81%) with greater sequence divergence in the C-terminal residues (11%–25% identity). HER2 appears to be the preferred binding partner for the formation of heterodimers with other members of the ERB family. The HER2/EGFR heterodimer shows enhanced potency when compared to the EGFR homodimer [10]. The HER2 kinase is intrinsically autoinhibited in the cell unlike other receptor tyrosine kinases that require phosphorylation of the activation loop. Recent research on the kinase domains of the dimers has revealed an allosteric mechanism of activation of EGFR and HER4 via dimer formation [38,39,40,41].

### 3.1. HER2 Tyrosine Kinase Inhibitors

#### 3.1.1. Lapatinib

**Figure 1:**
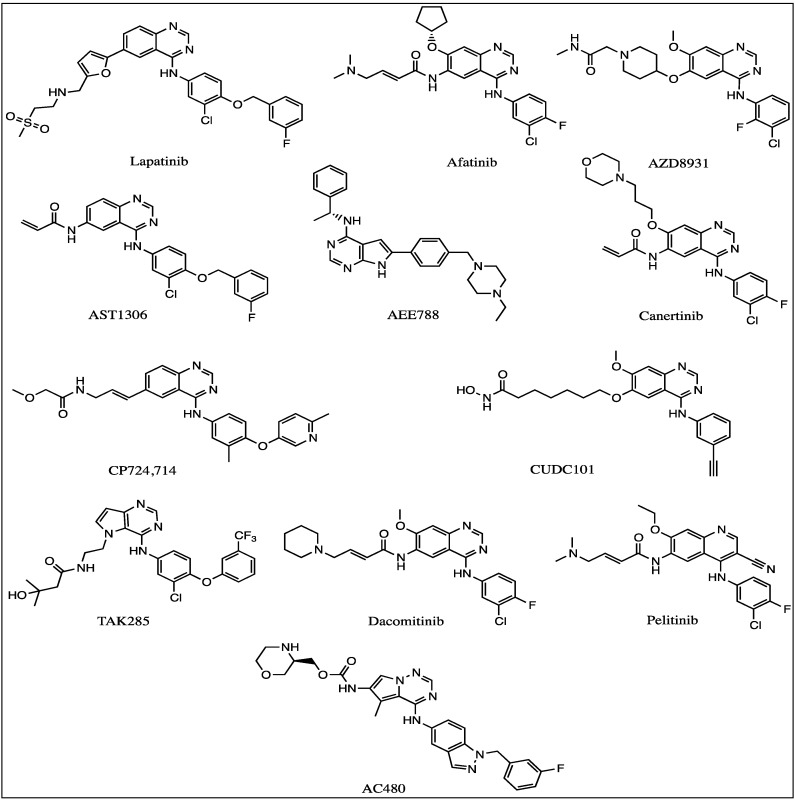
Structures of the HER2 tyrosine kinase inhibitors.

Lapatinib (Tykerb/Tyverb**^®^**) (Figure 1) is an orally active dual EGFR/HER2 tyrosine kinase inhibitor. The development of lapatinib was determined in part by the preclinical evaluations favoring synergistic cell proliferation inhibition using EGFR targeted therapies alongside HER2 inhibitors [11,42]. Lapatinib demonstrated a greater apoptotic response than single agent inhibition alone in SKBR-3 and BT-474 HER2 overexpressing cell lines [43].

In a recent randomized phase III trial evaluating the efficacy of dual targeted trastuzumab and lapatinib treatments in early-stage HER2 positive breast cancer, the North American Breast Cancer Group (NABCG) and the Breast International Group (BIG) found no significant statistical advantage in invasive disease free survival over single agent trastuzumab treatment alone in an 8000 patient population size [25,44]. The Adjuvant Lapatinib and/or Trastuzumab Treatment Optimisation (ALTTO) trial compared patients receiving a combination of three lapatinib-containing regimens with trastuzumab over the course of a year. Only 6.9% of patients with disease free survival using lapatinib and trastuzumab dual therapies were observed over a 4.5-year median follow up. The study, while groundbreaking in sheer population size and diversity, was somewhat disappointing in the potential of lapatinib as a combinatorial therapeutic agent with trastuzumab. Nevertheless, the data will provide insight into a more comprehensive understanding of cooperative drug inhibition and enable better treatment options for current and future HER2-positive patients.

#### 3.1.2. Afatinib

Afatinib (Gilotrif/Giotrif**^®^**) is a highly selective orally bioavailable irreversible pan inhibitor approved for use in treating metastatic non-small cell lung carcinoma (NSCLC). While its antitumoral activity is well documented in NSCLC, its activity targeting metastatic breast cancer is still being evaluated. Afatinib has an *in vitro* IC_50_ of 0.5 nM in EGFR and 14 nM in HER2 and showed promising activity in preclinical studies using EGFR and HER2-overexpressing trastuzumab-resistant cell lines (SUM 190-PT) and HER2-negative cells (SUM 149-PT) [45,46,47]. It also demonstrated encouraging results in multiple phase I clinical trials when used as a monotherapy and in combination with chemotherapy though its toxicity profile remains high [46,48,49].

#### 3.1.3. AZD8931

AZD8931 is an orally active reversible equipotent inhibitor of EGFR, HER2, and HER3. It has shown to be more potent than lapatinib and gefitinib in NSCLCs and also exhibits high selectivity for HER kinases against those outside the HER family [43,50]. In a study investigating the antitumor activity of AZD8931 in preclinical models of EGFR-overexpressed and HER2 non-amplified breast cancer cell lines (SUM 149 and FC-IBC-02, respectively), significant suppression of cell growth and induced apoptosis was observed in combination with paclitaxel therapy [51]. A two-part phase I trial assessing the safety and tolerability of single agent AZD8931 in patients with advanced solid tumors and as a combinatorial therapy with paclitaxel in female patients expressing advanced metastatic breast cancer showed no dose-limiting toxicities in either case [52]. AZD8931 was highly absorbed (median t_max_ = 1–3 h) in another study, displaying an elimination half-life of approximately 11 hours with moderate to high clearance; while the maximum tolerated dose from a 21-day evaluation was 240 mg [53]. However, more data must be obtained to confirm an appropriate maximum tolerated dose for use in chronic treatment.

### 3.2. Emerging HER2 Tyrosine Kinase Inhibitors

#### 3.2.1. AST-1306

AST-1306 is a selective orally active irreversible EGFR and HER2 inhibitor. Studies demonstrated weakly inhibiting EGFR tumor suppression activity in SK-OV-3 cell lines when HER2 knockdown occurred *in vivo*, and potent tumor suppression was exhibited in HER2-overexpressing adenocarcinoma xenograft and FVB-2/N(neu) transgenic breast cancer mouse models [54]. A phase I dose-escalation study evaluating the safety, tolerability, pharmacokinetics, and anti-tumor effects of AST-1306 found it to be rapidly absorbed with moderate to high clearance; and a pharmacokinetic concentration consistent over the evaluated dose range [55]. Further data is needed, but a recommended dosage of a 1000 mg three times daily is suggested for additional phase II trials [55].

#### 3.2.2. AEE-788

AEE-788 is a dual specific reversible EGFR and HER2 kinase inhibitor. A study investigating the effects of AEE-788 on EGFR and HER2 in glioblastoma cells (LN-18 and LN-229 cell lines, respectively) expressing varying co-expression levels followed by experimental radiotherapy showed HER2-overexpressing sensitivity, while demonstrating induced inhibition on previously resistant EGFR-overexpressing cells [56]. AEE-788 also showed EGFR/HER2 heterodimerization inhibition in overexpressing LN-18 cells, suggesting HER2 inhibition in EGFR-overexpressing cells may provide a therapeutic strategy in EGFR resistant cells. The antitumoral efficacy of AEE-788 in combination with tamoxifen or letrozole showed enhanced anti-proliferative effects in estrogen receptor positive (ER+) cell lines (MCF-7 2A, ZR75.1 A3 and BT474 A3) [57]. However, when used as a single agent and in combinatorial endocrine therapy treatment, an elevation in progesterone receptor (PGR) and trefoil factor 1 (TFF1) gene expression was observed in BT474 A3 cells [57]. In combination with letrozole, AEE-788 was also found to produce significant inhibition compared to tamoxifen dual therapy in ZR75.1 A3 xenograft, suggesting AEE-788 and letrozole may provide increased tumor inhibition in breast cancer tumor cells when compared against single agent use or in combination with tamoxifen alone.

#### 3.2.3. CI-1033 (Canertinib)

CI-1033 is a highly selective irreversible orally bioactive EGFR, HER2, and HER4 tyrosine kinase inhibitor. CI-1033 was evaluated for its ability to inhibit tumor formation in esophageal squamous cell carcinoma (TT, TE2, TE6, TE10 cell lines) *in vitro* and *in vivo* with EGFR and HER2 overexpression in all four cell lines [58]. It was found to silence MAPK and Akt signaling pathways along with the suppression of kinase phosphorylation. As a single agent treatment in a randomized phase II trial evaluating patients with pretreated metastatic breast cancer, CI-1033 showed no meaningful clinical activity. However, antitumor activity was observed in one arm of the study, though doses higher than 50 mg were generally not well tolerated, and unacceptable toxicity levels were exhibited at the highest dose [59].

#### 3.2.4. CP-724714

CP-724714 is a reversible orally active selective HER2 kinase inhibitor. Early stage pharmacologic characterization studies showed CP-724714 to be a potent autophosphorylation inhibitor and G1 cell cycle blocking inducer in HER2-overexpressing BT474 human breast carcinoma cells [60]. It also demonstrated potent inhibition of HER2-overexpressed tumor growth in athymic mice with no signs of adverse effects. A phase I dose-escalating study evaluating the safety, tolerability, and pharmaco-kinetic effects on patients with advanced malignant solid HER2 expressing tumors found a maximum tolerated dose of 250 mg three times daily with a dose-limiting toxicity including elevated alanine aminotransferase, thrombocytopenia, and hyperbilirubinemia as well as pulmonary embolus [61]. It was proposed that CP-724714 induced inhibition of hepatic efflux transporters that contributed to an accumulation of drug and bile levels in the liver leading to hepatobiliary cholestasis [61]. CP-724714 has since been discontinued in clinical development.

#### 3.2.5. CUDC-101

The discovery of CUDC-101, an irreversible HDAC, EGFR, and HER2 inhibitor, resulted from the incorporation of histone deacetylase (HDAC) functionality into the EGFR and HER2 inhibitor pharmacophore. It showed higher potency than erlotinib and lapatinib in most of the tumor lines tested, with an EGFR and HER2 *in vitro* kinase IC_50_ of 2.4 and 15.7 nM respectively [62]. Due to HDAC action, CUDC-101 was shown to reduce regulation in multiple proliferative signaling pathways such as MET, Akt, and HER3, allowing it to overcome some limitations brought about by conventional HER signaling inhibition [63]. A phase I trial investigating the maximum tolerated dose in patients receiving chemoradiation in untreated locally advanced squamous cell head and neck carcinomas found a maximum dose of 275 mg/m^2^ with a dose limiting toxicity of acute renal failure derived from elevated creatinine levels in one patient [64].

#### 3.2.6. TAK-285

TAK-285 is an orally active irreversible potent dual EGFR/HER2 inhibitor exhibiting an IC_50_ of 2.5 and 0.98 nM, respectively, and a cell growth inhibitory activity (GI_50_) of 2.0 nM in subcutaneous mouse BT-474 cells. It also showed potent tumor regression in both EGFR-overexpressing (4-1ST) and HER2-overexpressing (CAL27) tumor xenograft mouse models with 50 mg/kg and 100 mg/kg oral doses [65]. Its therapeutic potential in HER2-overexpressing brain metastasis was verified by *in vivo* microdialysis evaluations detecting unbound levels of TAK-285 in the extracellular space in the brain for up to 24–48 h after administration [66]. The toxicity profile and pharmacokinetic studies in a phase I trial found rapid absorption after oral dosing with increased plasma exposure in a dose-dependent fashion. The dose limiting toxicities were noted as a grade 3 increase in aminotransferases as well as grade 3 appetite suppression in a 400 mg twice daily regimen [67].

#### 3.2.7. AC-480 (BMS-599626)

AC-480 is an orally bioavailable reversible EGFR, HER2, and HER4 inhibitor. It showed an accumulation of cells in the G1 phase and demonstrated significant enhancement of radiosensitivity in NH-5 cells, as well as phosphorylation inhibition of EGFR, HER2, Rb, Akt, MAPK, CDK1/2/6, and Ku70 proteins [68]. In a phase I trial in patients with advanced solid tumors, a maximum tolerated dose of 600 mg/day was recommended, with a dose-limiting toxicity of grade 3 elevated hepatic transaminases and QTc interval prolongation reported in 660 mg/day doses [69]. AC-480 was well tolerated in the phase I study with 11 patients (*n =* 45) exhibiting a stable disease condition of 4 months or greater. It is also of note that AC-480 exhibited increased cell line-dependent synergistic or additive apoptosis in head and neck squamous cell carcinomas when used as a co-inhibitor with the insulin-like growth factor-1 receptor 1 (IGF1R) inhibitor, BMS-754807 [69].

#### 3.2.8. PF299804, PF299 (Dacomitinib)

Dacomitinib is an orally active irreversible EGFR, HER2, and HER4 inhibitor. A phase I dose-escalation study in patients with advanced NSCLC solid tumors found dacomitinib to be well tolerated, exhibiting a maximum tolerated dose of 45 mg/day and a dose-limiting toxicity of stomatitis and skin toxicities [70]. Patients previously treated with erlotinib or gefitinib showed the most encouraging signs of antitumor activity. Dacomitinib also demonstrated inhibition of HER2-amplified trastuzumab and lapatinib resistant breast cancer cell lines *in vitro*; exerting its antiproliferative effects through a G0- G1 combined cell cycle arrest and inducing apoptosis [71]. A phase II trial investigating the effects of dacomitinib in patients with NSCLC who previously received failed erlotinib and chemotherapy treatments (*n =* 66) showed acceptable tolerability and limited objective response rates in adenocarcinoma (5%, *n =* 50) and nonademocarcinoma (6%, *n =* 16) patients [72].

#### 3.2.9. EKB-569 (Perlitinib)

Perlitinib is an orally active irreversible selective dual EGFR/HER2 inhibitor. A phase I dose-escalation trial investigating the administration of pelitinib in patients with advanced solid tumors found a maximum tolerated once daily dose of 75 mg/day, with a low toxicity profile showing grade 3 diarrhea as the major dose-limiting toxic effects [73]. No significant antitumor responses were observed, however one NSCLC patient and another cutaneous squamous cell carcinoma patient showed stable disease for 33 and 24 weeks, respectively (Table 2).

**Table 2:** Specificity profiles of HER2 kinase inhibitors.

Inhibitor	EGFR IC_50_ (nM)	HER2 IC_50_ (nM)	HER3 IC_50_ (nM)	HER4 IC_50_ (nM)	Characteristics
Lapatinib [74]	10.2	9.8	-	367	Reversible
Afatinib [75]	0.5	14	-	-	Irreversible
AZD8931 [50]	4	3	4	-	Irreversible
AST-1306 [54]	0.5	3.0	-	0.8	Reversible
AEE-788 [76]	2	6	-	160	Reversible
CI-1033 (Canertinib) [77]	1.5	9.0	-	-	Irreversible
CP724,714 [60]	-	9.8	-	-	Reversible
CUDC-101 [78]	2.4	15.7	-	-	Irreversible
TAK-285 [79]	24	36	-	260	Irreversible
AC-480 (BMS-599626) [80]	22	32	-	190	Reversible
PF299804 PF299 (Dacomitinib) [75]	6.0	45.7	-	73.7	Irreversible
EKB-569 (Pelitinib) [73]	38.5	1.26	-	-	Irreversible

**Figure 2:**
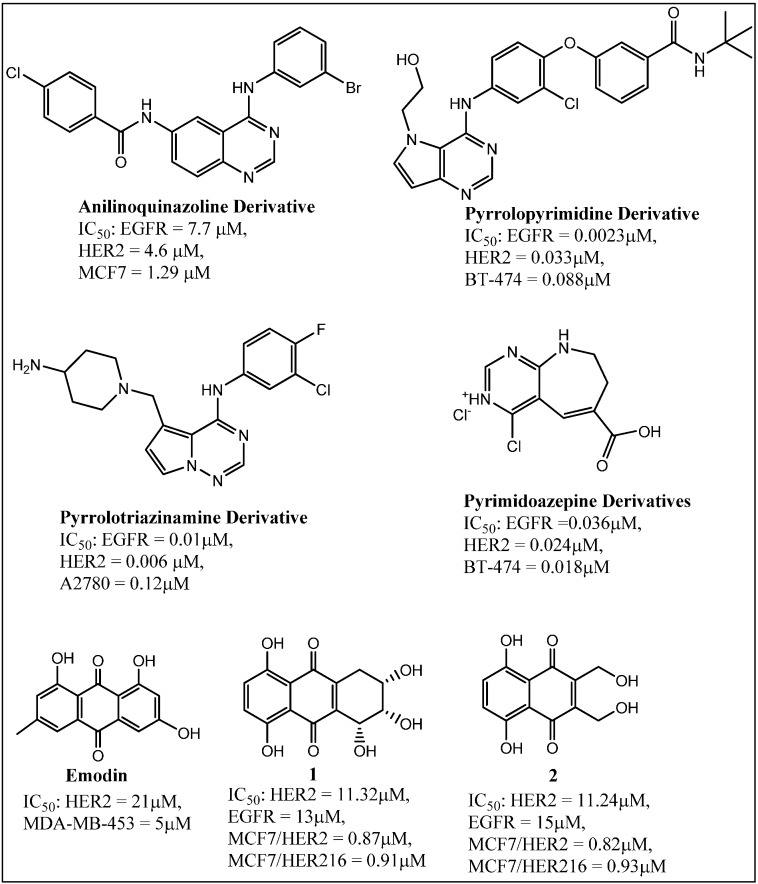
Structures of new HER2 kinase inhibitors.

### 3.3. Ongoing Search for New Inhibitors

Several classes of organic molecules are being explored for their potential to inhibit EGFR and HER2 tyrosine kinases. Among them several nitrogen heterocycle-containing scaffolds such as pyrrolotriazinamines [81], anilinoquinazolines [82,83], pyrimidoazepines [79], pyrrolopyrimidines [84,85], and purines [86] have been found to inhibit EGFR and/or HER2 kinase with significantly high potency. The structures of some of the most potent inhibitors are given in Figure 1. Other than nitrogen heterocycles as tyrosine kinase inhibitors, anthraquinones such as emodin [87] and quinone derivatives analyzed in our laboratory [88] have shown the potential to inhibit the growth of breast cancer cells through inhibition of the tyrosine kinases belonging to the ERB family of receptors. Emodin is known to be a pan kinase inhibitor. The ability of the compounds to inhibit HER2 kinase activity directly was tested by using the ADP-Glo *in vitro* kinase assay kit from Promega. The quinones **1** and **2** (Figure 2) inhibited EGFR and HER2 kinases with equal but low micromolar potency. But these same quinones showed a much higher potency in the inhibition of breast tumor cells expressing HER2 or the trastuzumab resistant HER2 oncogenic isoform, HER2Δ16 (The IC_50_ of the effective compounds was measured by treating each of the cell lines with different drug concentrations for 48 h followed by the CellTiter-Glo Assay to detect cell viability).

## 4. Conclusions

The development of resistance to monoclonal antibody trastuzumab within a year of treatment and the presence of truncated forms of HER2 (p95^HER2^ and HER2Δ16) has led the shift in focus of research to the targeting of the kinase domain for the development of therapeutics. Even though lapatinib, a dual inhibitor of EGFR and HER2 kinases showed great promise in phase I and phase II trials, a subsequent phase III trial found no significant advantage over the single agent treatment trastuzumab in disease free survival. Many other small molecule inhibitors of the kinases are presently undergoing phase trials. While some have shown toxicity leading to the discontinuation of their phase trials, other agents have moved on to the phase III trials. The therapeutic potential of these new agents is still unproven. The need remains for a therapeutic agent that can have a long term impact on the treatment of breast cancer and to ensure disease free survival.
